# IMP1, an mRNA binding protein that reduces the metastatic potential of breast cancer in a mouse model

**DOI:** 10.18632/oncotarget.12083

**Published:** 2016-09-16

**Authors:** Chiso U. Nwokafor, Rani S. Sellers, Robert H. Singer

**Affiliations:** ^1^ Department of Anatomy and Structural Biology, Albert Einstein College of Medicine, Bronx, NY, USA; ^2^ Department of Pathology, Albert Einstein College of Medicine, Bronx, NY, USA

**Keywords:** cancer, breast cancer, metastasis, RNA binding protein, IMP1

## Abstract

Cells that are able to localize β-actin mRNA efficiently have decreased metastatic potential. Invasive carcinoma cells derived from primary mammary tumors have reduced levels of an RNA binding protein IMP1/ZBP1/IGF2BP1, required for β-actin mRNA localization. We showed previously that in human breast carcinoma cells in vitro, this protein suppresses invasion. In this work we examined whether its re-expression can suppress breast cancer metastasis in a breast cancer mouse model. We developed a mouse conditionally expressing IMP1-GFP (hereinafter referred to as the IMP1 transgene) specifically in the mammary gland of a PYMT breast cancer mouse. We found that mice conditionally expressing the IMP1 transgene showed little or no metastases to the lungs from the primary tumor in contrast to PYMT mice not expressing IMP1, which uniformly develop metastases at an early stage.

## INTRODUCTION

Spreading of malignant cells from a primary tumor to other parts of the body is a leading cause of morbidity and mortality in cancer patients and breast cancer metastasis is the leading cause of death in women [1]. Understanding how cancer spreads from the primary tumor is critical for the development of new diagnostic tools and therapies. Metastatic cells are responsive to chemotactic signals and therefore orient and move efficiently towards blood vessels [2]. Thus alterations that increase the cell's migratory capacity are the key in the transition to a metastatic phenotype.

Human IMP1 (IGF2 mRNA-binding protein 1) originally cloned as ZBP1 [3], is an important RNA-binding protein important for cell RNA localization, migration, stability, and translational control. It facilitates the localization of β-actin, E-cadherin, α-actinin and ARP2/3 complex mRNAs, which are involved in cell-cell connections and focal adhesions [4]. Loss of IMP1 increases the growth ability of metastatic cells and promotes cell motility but decreases persistence [5, 6]. Undirected cell motility is required for tumor cell spreading and subsequent invasion of connective tissue, lymphatic and blood vessels [7]. IMP1 is expressed during embryogenesis, but expression of IMP1 is reduced in most adult tissues [8]. We have previously shown that the IMP1 promoter binds to β-catenin, a protein that is involved in both cell adhesion and transcription [8]. In breast cancer cells, the expression of IMP1 and the expression of β-catenin are coordinately regulated [8].

IMP1 gene reactivation has been observed in various human tumors [9]. Because of its association with and modulation of various mRNAs associated with cell motility and adhesion, changes in IMP1 expression may affect the metastatic potential of cancer cells. IMP1 has been shown to have a suppressive effect on the proliferation and metastasis of breast cancer cells. IMP1 has a negative effect on metastasis when re-expressed in non-IMP1 expressing cells. Re-expression of IMP1 in MTLn3 cell lines derived from a mouse breast carcinoma that lacks IMP1 expression inhibited lung metastasis of the cells from the primary breast tumor [10]. In a colon cancer model, loss of IMP1 function in stromal cells provided a microenvironment that enabled tumorogenesis [11]. A more recent study in mice [10] showed that IMP1 suppressed breast tumor formation and lung metastasis derived from the growth of an implanted human MDA231 breast cancer cell line.

To further investigate whether IMP1 can suppress metastasis in a known breast cancer mouse model *in vivo*, doxycycline inducible IMP1-GFP transgenic mice [12] were generated since endogenous IMP1 expression in adult tissue is mostly silenced. These tags have been shown to have no effect on the RNA-binding of IMP1 protein [13]. Our results demonstrate that this transgene suppresses metastases to the lung.

## RESULTS

### Detection of transgenic IMP1 expression in MMTV-PYMT-IMP1 mice

In order to evaluate the role of IMP1 in breast cancer metastasis, we crossed the MMTV-PyMT breast cancer mouse model with a conditionally expressing IMP1-GFP transgenic mouse (10). The PyMT oncogene is driven by the MMTV promoter [8]. PyMT is a viral oncogene that when overexpressed in the mouse mammary gland causes widespread proliferation of the mammary epithelium which progresses to multifocal mammary adenocarcinomas and pulmonary metastasis [15]. This mammary cancer mouse model is characterized by short latency, high penetrance, and a high incidence of lung metastasis [16].

**Figure 1:**
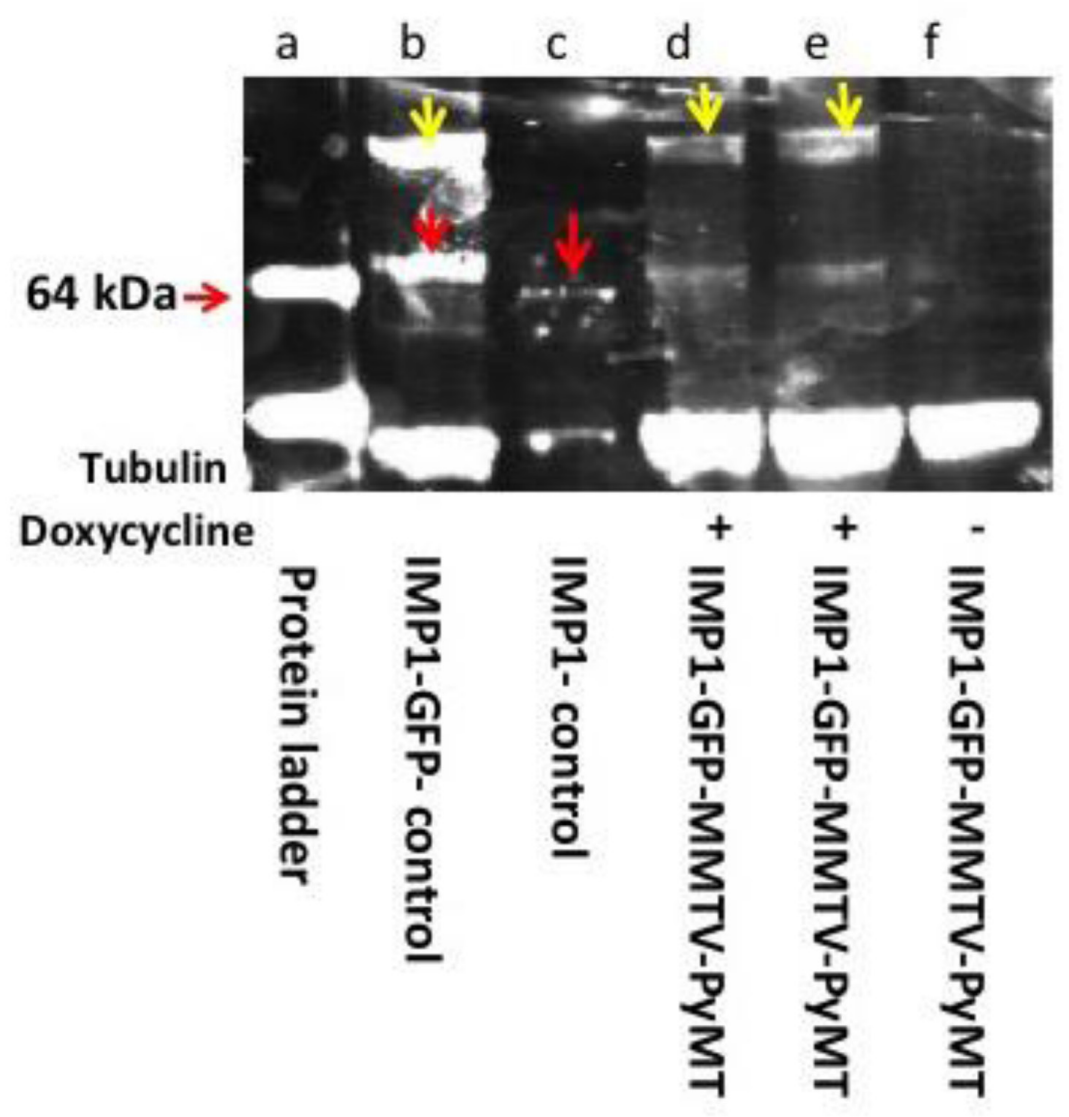
IMP1-GFP fusion protein detection Western blot analysis of MMTV-PyMT-IMP1 tumors + and - doxycycline induction using polyclonal anti-IMP1 antibodies. Lane a, protein molecular weight markers. Lane b, IMP1-GFP. 91KDa, isolated from cultured mouse embryonic fibroblasts (yellow arrows), endogenous IMP1, 64kDa (red arrows). Lane c, endogenous IMP1 band (red arrow) from cultured MEFs. Lanes d and e mammary tumor lysates from MMTV-PYMT mice expressing the transgenic IMP1-GFP fusion protein (yellow arrows) + doxycycline induction. Lane f tumor lysates from MMTV-PYMT-IMP1 mice - doxycycline induction (IMP1-GFP not expressed).

The MMTV-PyMT-IMP1 transgenic mice express IMP1 protein upon induction using 1mg/ml of doxycycline. We used a specific IMP1 antibody generated in our laboratory to detect IMP1 protein expression. We validated the inducible expression of the transgene in fibroblasts cultured from the mice as well as from tumor lysates. There was no detectable IMP1 expression in mammary tumors from MMTV-PYMT-IMP1 mice without doxycycline induction (Figure 1).

### Effect of transgenic IMP1 expression on tumor formation and mammary tumor burden

**Figure 2:**
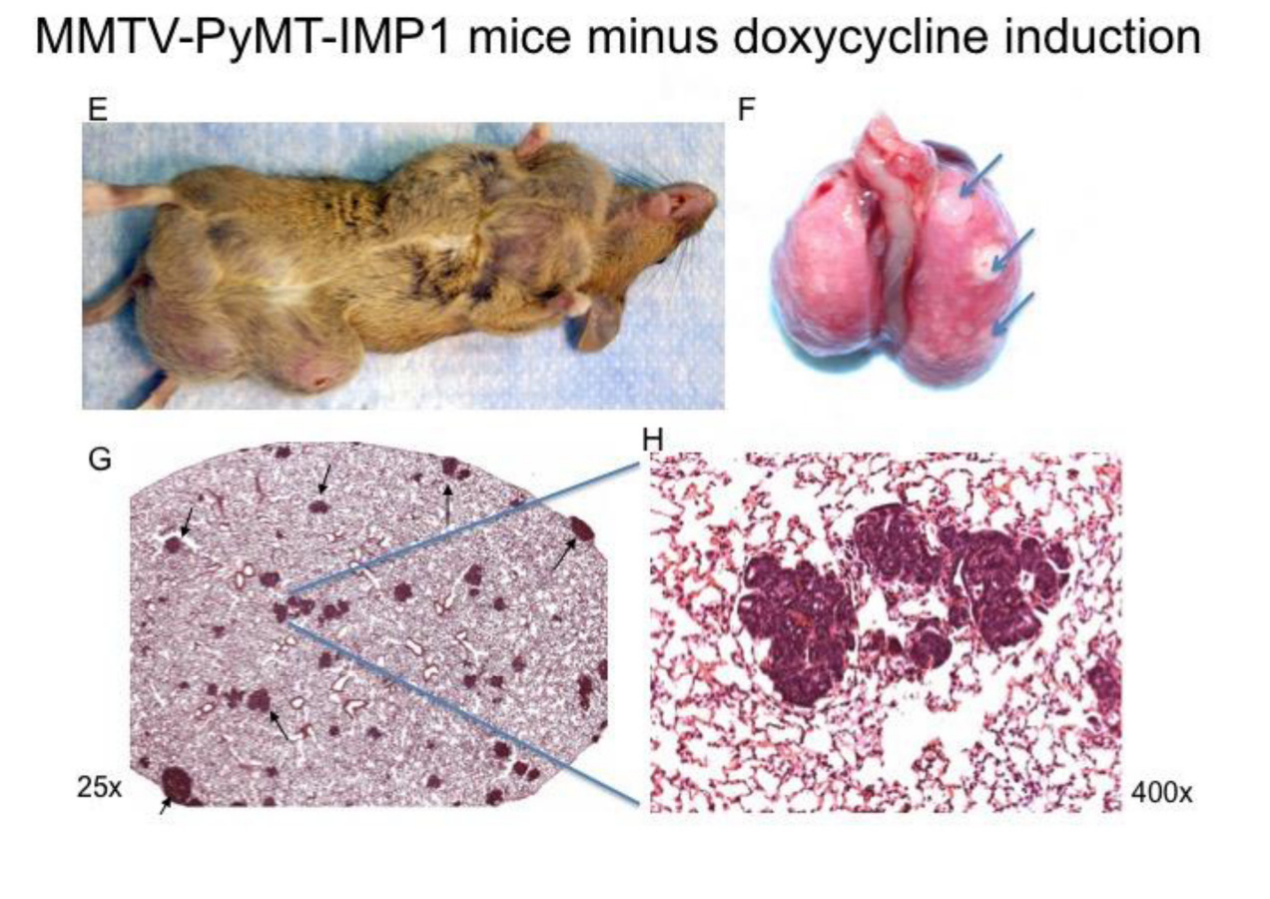
Gross observation of the primary mammary tumor, and H&Es of lung metastasis **A.** MMTV-PYMT-IMP1 mice plus doxycycline induction (*n* = 7 mice) develop mammary tumors, one representative mouse is shown. **B.** Gross analysis of whole lungs from these mice show either few or no lung metastasis (*n* = 7 lungs), one representative whole lung is shown. **C.** & **D.** H&E stains from representative lungs sections (*n* = 7 mice) show either few or no lung metastases. MMTV-PyMT-IMP1 mice (*n* = 7) minus doxycycline show more metastasis to the lungs. **E.** MMTV-PyMT-IMP1 mice (arrows, *n* = 7) minus doxycycline develop mammary tumors, one representative mouse is shown. **F.** Representative whole lungs with metastatic lesions (*n* = 7 lungs). **G.** & **H.** H&E stains from representative lung sections (*n* = 7 mice) show numerous lung metastases.

To determine the effect of IMP1 expression on tumor formation, we placed IMP1-GFP transgenic mice without MMTV-PyMT transgenes on doxycycline. Doxycycline treated IMP1-GFP mice without the MMTV-PYMT transgene (*n* = 22 mice) did not develop mammary tumors within the observation period (8 weeks) whereas all MMTV-PYMT transgenic mice with or without the IMP1 transgene induction developed multiple mammary gland masses (*n* = 18 mice). Examples are shown with two mice in Figure 2A & 2E. Mice were sacrificed when any individual tumor reached ~2 cm, which was generally 16 weeks after the first mass was identified. MMTV-PYMT-IMP1 mice (*n* = 7) with doxycycline induction developed mammary tumors but showed few or no lung metastasis (*n* = 7 mice, 7 lung sections Figure 2B and 2H & 2E stain of a representative lung section in Figure 2C & 2D). MMTV-PYMT-IMP1 mice (*n* = 7) without doxycycline induction developed mammary tumors but also developed pulmonary metastasis (*n* = 7 mice, *n* = 7 sections) based on the criteria defined by Lin et al., 2003 [16]. An example is shown with whole lung in Figure 2F and an H&E stain of a lung section in Figure 2G &2H.

**Figure 3:**
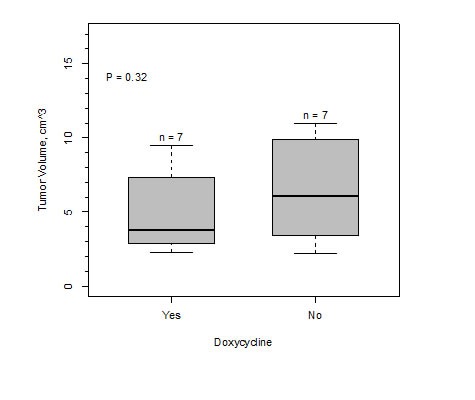
Bar graph of mammary tumor burden from MMTV-PYMT-IMP1 mice (*n* = 7 mice) plus doxycycline and MMTV-PYMT-IMP1 mice minus doxycycline (*n* = 7 mice).

The expression of IMP1 did not affect tumor growth or size. Table 1 shows the individual tumor sizes (lxhxw) and total tumor volumes per mouse for MMTV-PyMT-IMP1 mice with and without IMP1 induction by doxycycline. Analysis of the data by the Welch Two Sample *t*-test (*P* = 0.32) and one way anova (*P* = 0.35) demonstrated that there was not a significant difference in the means of total tumor volumes of both groups. Bar graph of tumor volume is shown in Figure 3.

**Table 1:** Tumor measurement (cm) per MMTV-PyMT-IMP1 mouse.

**Mouse ID**	1	2	3	4	5	6	7	8	9	10	11	12	13	14
**Doxycycline**	+	+	+	+	+	+	+	-	-	-	-	-	-	-
	3.2	2.6	5.8	7.8	4.8	2.6	5.3	7.3	3.0	5.8	4.5	9.0	5.5	3.6
	1.6	4.5	2.9	0.9	2.4	2.7		4.0	2.3	2.8	2.8	3.1	2.9	3.9
	2.4	3.4	2.4	0.9	2.2	2.9			3.1	4.6	3.4	2.4		5.7
		7.0	3.6		2.7	2.5			3.0	3.8	1.2	3.0		2.1
					3	3.3			2.6	5.6	0.6	5.3		2.1
					2.4	1.8				2.8	2.0	6.1		1.5
					2.5	1.5					5.0			
					4.1									
**Tumor Burden**	7.2	17.5	14.7	9.6	24.1	17.3	5.3	11.3	14.0	25.4	19.5	28.9	8.4	18.9

### Expression of the IMP1 transgene limits pulmonary metastases

**Figure 4:**
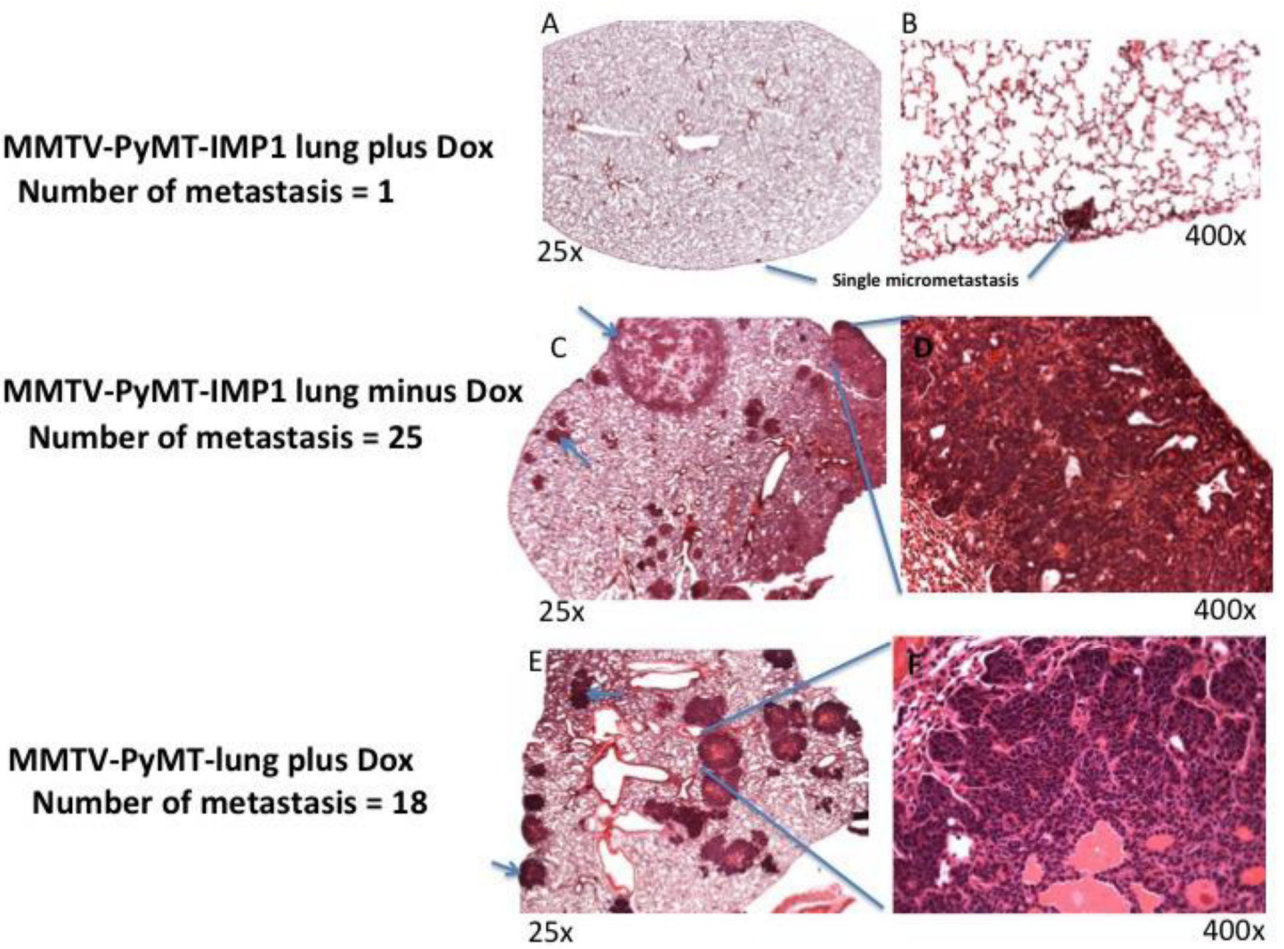
H&E stains of MMTV-PyMT-IMP1 lungs Photomicrographs of hematoxylin and eosin stained lung sections. A,C&E (magnification, 25x) and B,D&F (magnification, 400x). **A.** & **B.** Representative lung section (*n* = 7 mice and 7 representative lung sections) from MMTV-PyMT-IMP1 mouse plus doxycycline. **C.** & **D.** Representative lung section (*n* = 7 mice and 7 representative lung sections) from MMTV-PyMT-IMP1 mice minus doxycycline. **D.** & **E.** Representative lung section (*n* = 5 mice and 5 representative lung sections) from MMTV-PyMT plus doxycycline in the absence of the IMP1-GFP transgene.

Despite the lack of an effect of IMP1 on tumor size, the ability of the cells to spread was profoundly affected. We performed histological evaluations on H&E stained lung sections to determine the presence and number of metastasis formed in all areas of the lungs in mice under various treatments. MMTV-PYMT mice without the IMP1 transgene and MMTV-PYMT-IMP1 mice without induction of the IMP1 transgene by doxycycline developed either gross or histological pulmonary metastatic disease (Figure 4C &4D*n* = 7 mice, *n* = 7 lung sections).

The metastases were typically large and multifocal, whether or not treated with doxycycline. Representative examples are shown with one 5μm lung section in Figure 4E & 4F (*n* = 5 mice, *n* = 5 lung sections; *P* = 0.034). In contrast MMTV-PYMT-IMP1 transgenic mice with IMP1 transgene induction by doxycycline (*n* = 7 mice, *n* = 7 lungs sections) did not present gross evidence of pulmonary metastases (*P* = 0.027). An example from one of these mice is shown in Figure 4A & 4B, indicating that IMP1 expression had a negative effect on metastasis.

**Figure 5:**
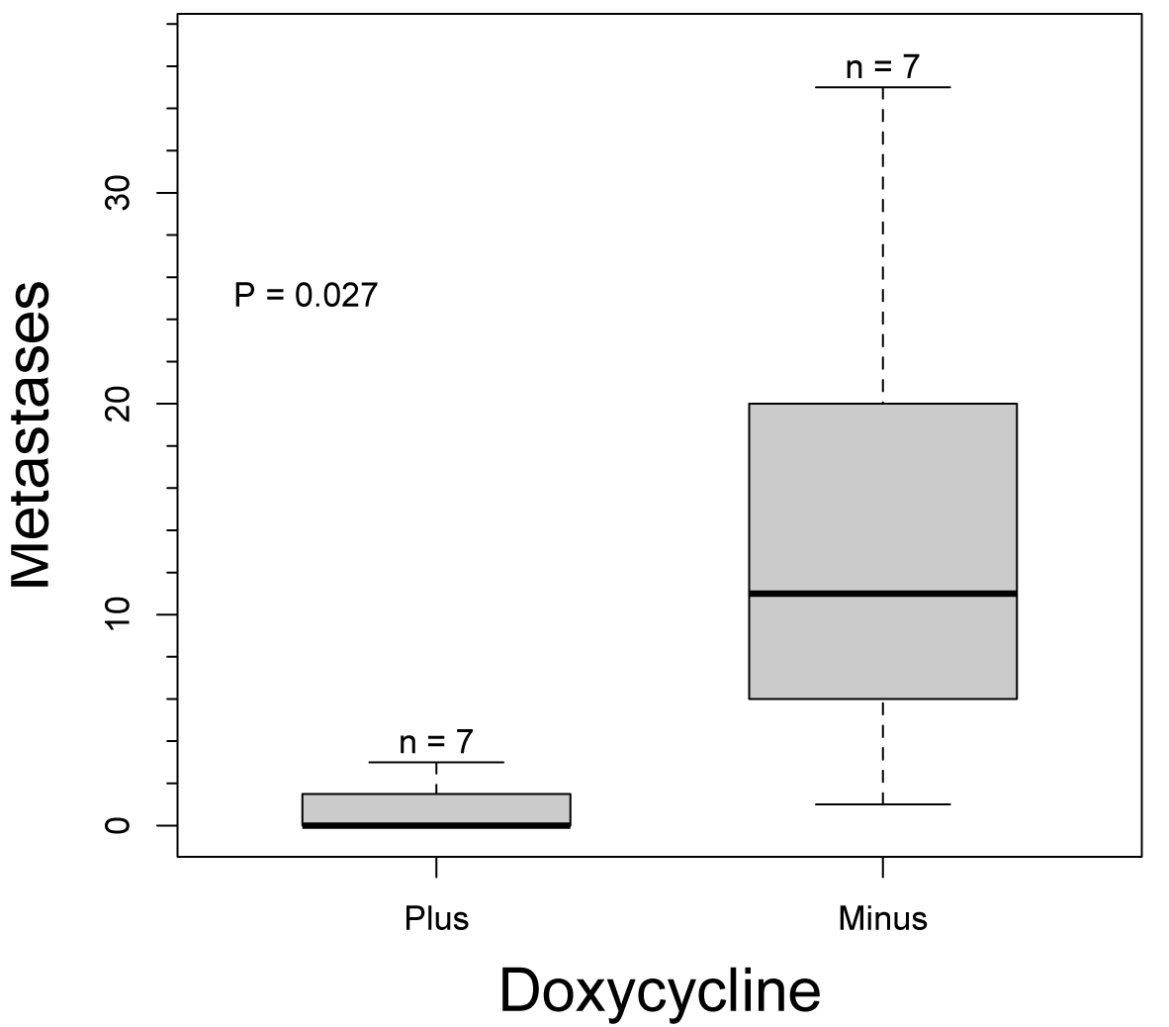
Effect of IMP1 transgene expression on pulmonary metastases Number of metastasis in MMTV-PyMT-IMP1 mice (*n* = 7 mice per group) plus and minus doxycycline.

To confirm the negative effect of IMP1 transgene expression on pulmonary metastasis in MMTV-PyMT breast cancer mouse model, we performed both a one way anova analysis and Welch Two Sample *t*-test (*P* = 0.027), both analysis show that there is a statistical significance in the negative role of IMP1 transgene expression on the incidence of pulmonary metastasis between MMTV-PyMT-IMP1 mice (*n* = 7 mice, *n* = 7 lungs sections) expressing the IMP1 transgene and MMTV-PyMT-IMP1 mice (*n* = 7 mice, *n* = 7 lung sections) not expressing the IMP1 transgene (Figure 5). We quantified metastasis using one representative 5μm lung section per mouse that had the most metastatic lesions.

## DISCUSSION

Previous studies have shown that expressing IMP1 could enhance persistence and directionality [17] and induce polarity in breast tumor cells in culture [4, 18]. It has been reported that IMP1 expression is decreased in a subpopulation of cells derived from invasive tumors and elevated in noninvasive cells from the same tumor [19]. Our current study extends these *in vitro* studies to a mouse breast cancer model. In order to do this we crossed the doxycycline inducible IMP1 transgenic mouse with the MMTV-PYMT breast cancer mouse model for *in vivo* experiments. Although some studies have shown that doxycycline decreases tumor burden in bone metastasis [2], we didn't observe any obvious effect of doxycycline on primary breast tumors.

All experimental and control mice used were in the same genetic background. All mice produced tumors but we found that when the breast carcinoma cells expressed the IMP1 transgene, the incidence of metastasis to the lungs from the primary tumor was significantly reduced. This *in vivo* observation suggested a role for IMP1 in suppressing breast cancer metastasis.

Progression to metastasis likely results in changes to the level of expression of certain genes including loss or gain of gene function [10]. Because the protein levels did not correlate exactly with mRNA levels, this suggested that IMP1 could be under translational control. IMP1 mRNA re-activation has been observed in human breast cancer, [9] and colon cancer, [20] as well as non-small cell lung carcinomas, [21]. It is important to note that some studies have shown that IMP1 protein levels were increased in a variety of cancers such as primary human malignant melanomas and in melanoma cell lines, [22]. IMP1 has also been shown to stimulate autoimmune responses in ovarian cancer [23]. In contrast to our study, expression of an IMP1 ortholog was previously reported to be increased in the progression of colorectal carcinoma to lymph node metastasis in patients [24]. These studies also showed that IMP1 protein was significantly reduced in breast cancer compared to colorectal cancer. Thus a likely reason why is that many studies using an RT PCR approach on patient samples have found IMP1 mRNA to be widely expressed in human breast tumors [10]. IMP3, another homolog of IMP1, has been shown to prevent miRNA-directed High-Mobility Group AT-hook 2 (HMGA2) mRNA decay in cancer and development, [25]. Re-expressing IMP1 has also been reported to cause mammary tumors [26], however these mammary tumors did not metastasize to the lungs [27]. In sum, our results are consistent with previous demonstrations *in vitro* and in cell culture that non metastatic mammary tumors express IMP1 and that invasive breast cancer metastatic cells have lower IMP1 levels [28]. Absence of IMP1 function increased growth ability of metastatic cells, decreased cell adhesion, and increased the ability of cells to respond to signals necessary for invasion [8] [18]. Because IMP1 binds a plethora of mRNAs involved in cell migration and proliferation, such as β-catenin, deregulation of these mRNAs when IMP1 is repressed could be a leading cause of the phenotypic changes observed in breast cancer cells, [5]. For instance, β-catenin binds the IMP1 promoter. IMP1 repression leads to deregulation of β-catenin, disrupting β-catenin signaling. Epigenetic methylation of the IMP1 promoter leads to its transcriptional inactivation because β-catenin is unable to bind the methylated IMP1 promoter. Thus inactivation of the IMP1 gene via promoter methylation induces the proliferation of metastatic cells. Previous studies showed that IMP1 was repressed in the following mammalian breast cancer cells; rat metastatic MTLn3 cells, [28], MDA 231 and 4T1 cells [5]. Consistent with their data, we observe that expressing the IMP1 transgene in mammary tumors of a breast cancer mouse model reduced the metastatic potential of the primary tumor.

There is an alternative explanation for the lack of agreement between IMP1 expression and progression of the disease. The re-expression of IMP1 in patients during cancer metastasis may occur in primary and secondary tumors, but be suppressed only in a few cells that metastasize. Once secondary tumors are formed, IMP1 may be re-expressed leading to the erroneous conclusion that it is associated with metastasis. One example supporting this view is exemplified by the Mena isoform 11a with high expression in primary and secondary tumors but a decrease in expression when cells are undergoing migration and metastasis. The isoform then re-expresses in the secondary tumor. Low levels of Mena 11a expression are predictive of metastasis in breast cancer patients, consistent with its suppression of invasion in breast tumor cells [29-31]. By analogy we predict that IMP1 is likewise repressed when cells are migrating from the tumor. Because the expression of IMP1 is needed to maintain cell adhesion [4], decrease in its expression would result in cells on the periphery of the tumor becoming less adhesive and the propensity to migrate away would become more likely.

Our study sheds light on two important aspects of breast cancer metastasis. First, our study supports the hypothesis that RNA binding proteins play a crucial role in breast cancer metastasis [32]. Second we show that increased levels of one of these proteins, IMP1 *in vivo*, can have an effect in the progression of breast cancer from late carcinoma to metastasis. Our animal study is in line with some in vitro, cell culture and xenograft studies that suggested IMP1 expression level has a negative role in cancer metastasis. Based on this study, the induction of IMP1 expression in breast cancer may be a potential therapeutic approach to prevent breast cancer progression. More *in vivo* experiments to better understand how IMP1 reduces the metastatic potential of the primary breast tumor and high throughput screening of small compounds that increase IMP1 expression in late mammary carcinomas will be important avenues for future study.

## MATERIALS AND METHODS

### Animals

Animal handling and use was in accordance with a protocol approved by the Animal Care and Use Committee of Albert Einstein College of Medicine and NIH guidelines. Up to five mice were housed per cage, and animals were maintained on a 14-h light: 10-h dark cycle with food and water available. Progeny of transgenic IMP1 mice have been back crossed more than four generations to C57BL/6J and were crossed with male transgenic mice expressing the PyMT oncogene under the control of MMTV LTR promoter, provided by Jiufeng Li and were bred in house. For experiments, transgenic IMP1 mice used in the study were heterozygous for IMP1 transgene, MMTVrtTA and the PYMT oncogene and were all aged matched. Control mice were litter mates that did not express the IMP1 transgene. Mice were treated with doxycycline at weaning, three weeks of age. For induction of IMP1, doxycycline (Clontech) was added to the water at 1 mg/mL in water bottles covered with aluminium foil to prevent direct exposure of doxycycline treated water to light. Doxycyline treated water was changed weekly until the tumors reached the maximum size allowed in the protocol approved by the Animal Care and Use Committee of Albert Einstein College of Medicine and NIH guidelines, after which mice were sacrificed, there were no obvious signs of dehydration in mice that were administered doxycycline.

### Genotyping

Genotyping of IMP1 transgenic mice was performed by Southern blot. Genomic DNA was isolated from tail tissue using the DNeasy kit from Qiagen. Tails were kept rocking at 55°C in 500 μL of tail buffer (100 mM Tris at pH 8.5, 5 mM EDTA, 0.2% SDS, 200 mM NaCl) with 100 μg/mL proteinase K overnight. Digestion was followed by vortexing and centrifugation for 15 min at 16,100g and precipitation of the supernatant with an equal volume of isopropanol. DNA was then removed and re-suspended by rocking overnight at 55°C in 100 μL of dH2O. After resuspension, DNA was precipitated before digestion with PvuII and gel loading (1% agarose with TBE). Gels were soaked while rocking in 0.25 M HCl for 10 min and rinsed three times in dH2O, followed by 40-min incubation while rocking in 0.4 M NaOH. Transfer onto Hybond N + (Amersham) membranes was performed in 0.4 M NaOH overnight, and blots were prehybridized at 42°C in 2× SSC, 1% SDS, 10% Dextran sulfate, 50% formamide for at least 1 h prior to hybridization at 42°C overnight. Blots were washed with 2X SSC, 1% SDS at 42°C for 10 min, followed by one to two washes with 2X SSC, 1% SDS at 65°C for 20 min, and then one to two washes with 0.2X SSC, 1% SDS at 65°C for 30 min before exposing to Kodak Biomax XAR film (Kodak). The 1.1-kb XhoI fragment of KL1448 was used as a probe for genotyping by Southern blotting. PCR was used to detect the PYMT transgene using JumpStart RED Taq Ready-mix from Sigma and a thermo cycler profile beginning with 3 min at 94°C, followed by 12 cycles of 94°C for 20 sec, 64°C for 30 sec, and 72°C for 35 sec, and then 25 cycles of 94°C for 20 sec, 58°C for 30 sec, and 72°C for 35 sec, and ending with 2 min at 72°C. PYMT forward primer, GGA AGC AAG TAC TTC ACA AGG G and reverse primer is GGA AAG TCA CTA GGA GCA GGG. rtTA-standard primers were used to detect the rtTA transgene using the same thermos cycler profile for the PYMT transgene. Primer 9009, GGC GAG TTT ACG GGT TGT TA. Primer 9116, CTG GTC ATC ATC CTG CCT TT. Primer oIMR8744, CAA ATG TTG CTT GTC TGG TG. Primer oIMR8745, GTC AGT CGA GTG CAC AGT TT.

### Western blots

Protein extracts were prepared from mammary gland tumors. Tumors were repeatedly washed in a wash buffer containing ice-cold PBS, 1 mM EGTA, 0.5 mM PMSF, and large chunks were fragmented after several washes and centrifugation of the tissue at 400g and 500g in the same wash buffer. Following all washes, an equal volume of lysis buffer (50 mM Tris at pH8.0, 150 mM NaCl, 5 mM MgCl2, 0.1 mg/mL Escherichia coli tRNA, 0.5 mM PMSF, 1 U/μL RNAseOUT (Invitrogen) 1× Complete Protease Inhibitor tablets (Roche), 0.5% NP40, 15 mM EDTA) was added to the tissue pellet and suspended. Samples were then incubated on ice for 5 minutes. Samples were again centrifuged for 10 min at 10,000g. The supernatant was separated from the pellet and centrifuged again to remove left over pellets. Protein in the supernatant was measured using a BCA assay (Pierce), and 5 or 10 μg in SDS sample buffer was loaded in each lane. The Invitrogen NuPAGE assay was used, and proteins were transferred onto Hybond ECL nitrocellulose membranes (Amersham) by wet blotting. Antibodies used were mouse anti-tubulin and a laboratory-prepared rabbit polyclonal anti-IMP1 raised against full-length His-tagged recombinant IMP1. Signal was visualized using the Odyssey Infrared Imaging System (Li-Cor) and analyzed using IPLab software (BD Biosciences).

### Histology

When any given mammary tumor was ~ 2 cm at its widest, tumor burden was calculated by adding the individual tumor volume per mouse. Mice were humanely euthanized and evaluated grossly and histologically. Primary tumors and the lungs were removed from mice. A sample of mammary gland tumor was frozen in liquid nitrogen for molecular evaluations and samples of primary tumor and lung were fixed for 48-72 hours in 10% neutral buffered formalin. Fixed tissues were routinely processed to paraffin, sectioned to a thickness of 5μm and stained with hematoxylin and eosin (H&E) for histological evaluation by a board certified veterinary pathologist using a Zeiss AxioSkop 2 light microscope. Photomicrographs were taken using a Zeiss AxioCam HRc and images were captured using Zeiss software. We quantified metastasis using one representative 5μm lung section per mouse that had the most metastatic lesions.

### Tumor measurements

In Figure 3, tumor volumes were obtained by measuring each tumor in length (l) x height (h) x width (w). Average radius (r) of each tumor was derived by l+h+w/6 and estimated volume was derived by 3.14 × 4/3 × r^3^ for each tumor. The total tumor volume for one mouse was obtained by adding the individual tumor estimate volumes.

In Table 1, total tumor burden was obtained by measuring each tumor in length (l) x height (h) x width (w) per mouse and volumes from each tumor were summed for each mouse.

### Statistical analysis

All statistical analysis and graphs were done in R (R Core Team (2015). R: A language and environment for statistical computing. R Foundation for Statistical Computing, Vienna, Australia. URL https://www.R-project.org/.)

## Abbreviations

PYMT: polyoma middle T virus
MMTV LTR: mouse mammary tumor virus long terminal repeat;
H&E: hematoxylin and eosin
BCA: bicinchoninic acid
rtTA: reverse tetracycline-controlled trans activator
PCR: Polymerase chain reaction
